# Spin–cavity interactions between a quantum dot molecule and a photonic crystal cavity

**DOI:** 10.1038/ncomms8665

**Published:** 2015-07-17

**Authors:** Patrick M. Vora, Allan S. Bracker, Samuel G. Carter, Timothy M. Sweeney, Mijin Kim, Chul Soo Kim, Lily Yang, Peter G. Brereton, Sophia E. Economou, Daniel Gammon

**Affiliations:** 1 NRC research associate residing at the Naval Research Laboratory, Washington, District of Columbia 20375, USA.; 2 Naval Research Laboratory, Washington, District of Columbia 20375, USA.; 3 Sotera Defense Solutions Inc., Columbia, Maryland 21046, USA.; 4 US Naval Academy, Annapolis, Maryland 21402, USA.

## Abstract

The integration of InAs/GaAs quantum dots into nanophotonic cavities has led to impressive demonstrations of cavity quantum electrodynamics. However, these demonstrations are primarily based on two-level excitonic systems. Efforts to couple long-lived quantum dot electron spin states with a cavity are only now succeeding. Here we report a two-spin–cavity system, achieved by embedding an InAs quantum dot molecule within a photonic crystal cavity. With this system we obtain a spin singlet–triplet Λ-system where the ground-state spin splitting exceeds the cavity linewidth by an order of magnitude. This allows us to observe cavity-stimulated Raman emission that is highly spin-selective. Moreover, we demonstrate the first cases of cavity-enhanced optical nonlinearities in a solid-state Λ-system. This provides an all-optical, local method to control the spin exchange splitting. Incorporation of a highly engineerable quantum dot molecule into the photonic crystal architecture advances prospects for a quantum network.

## Abstract (web summary)

Optical cavities enhance light–matter interactions, and have been used to strongly couple a photon to a single spin. Here, the authors take this a step further by coupling a photon to a two-spin system by embedding an indium arsenide quantum-dot molecule in a photonic crystal cavity.

## Graphical abstract

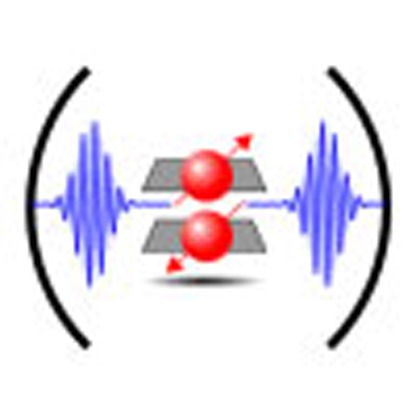

Much of the recent progress in developing photonic crystal cavities for quantum information is based on the two-level neutral exciton system in quantum dots (QDs)^1^. In contrast, cavity-based quantum information with single atoms takes advantage of a three-level Λ-system, consisting of two Zeeman levels and an excited state^2^. This approach has powerful advantages, yet the combination of solid-state Λ-systems with optical cavities is only in the early stages of exploration^3^,^4^,^5^,^6^,^7^. A limitation of QDs is that the Zeeman splittings are small compared with typical cavity linewidths. This makes it difficult to use the cavity for *selective enhancement* of one Λ branch, which is important for achieving spin–cavity interactions.

A solid-state cavity-Λ combination using QDs will provide both performance improvements and new capabilities, some of which have already been demonstrated. Complete qubit control with optical gates, initialization and readout has been performed on long-lived QD spins in a cavity, opening a potential route to scalability and a communication interface^4^. Raman spin–flip emission within this Λ-system has been demonstrated^6^, which can be used to convert spin states to photon states and as a source of tunable, indistinguishable single photons, although the spin selectivity so far is limited by the Zeeman splitting. Finally, cavity-enhanced quantum optical phenomena such as the Autler–Townes effect, coherent population trapping and the AC Stark effect are interesting in their own right as a means to study the fundamental quantum phenomena of the solid state, and they may provide the basis for controlling the quantum system and for quantum switch technologies^8^,^9^,^10^,^11^,^12^. Although recent work has demonstrated the AC Stark effect and the Mollow triplet in cavity-coupled two-level systems^13^,^14^,^15^,^16^,^17^, a strongly driven Λ-system in a cavity has not yet been studied.

In this work, we explore the full range of quantum optical phenomena that become possible when each branch of the Λ-system is selectively excited. We overcome the limitation of a small ground-state splitting by developing a cavity-coupled QD molecule (QDM; Fig. 1). QDMs (two QDs connected by a coherent tunnel barrier) have been studied in detail, but not yet in a cavity^12^,^18^,^19^,^20^,^21^,^22^,^23^,^24^,^25^,^26^,^27^,^28^,^29^,^30^,^31^. They have considerable design flexibility of both their optical and spin properties, which originates in the coherent tunnelling between QDs^21^,^25^. Here we use a QDM to obtain a Λ-system with a large spin singlet–triplet splitting even at zero-magnetic field. An applied bias charges the molecule with two electrons, and the resulting two-spin system has an exchange splitting of 1.45 meV, almost an order of magnitude larger than the 0.19 meV cavity linewidth. This is a new spectroscopic regime for cavity-coupled QDs, where the cavity can be coupled exclusively to either the excitation branch or the emission branch of the Λ-system. With cavity-coupled emission, we obtain much greater spin selectivity in the Raman process than with the one-spin system. Tuning the cavity to the excitation branch, we drive the QDM system strongly into the nonlinear regime to investigate the Autler–Townes state dressing and laser-induced control of the spin exchange energy.

## Results

### QDM in a cavity

**Figure 1:**
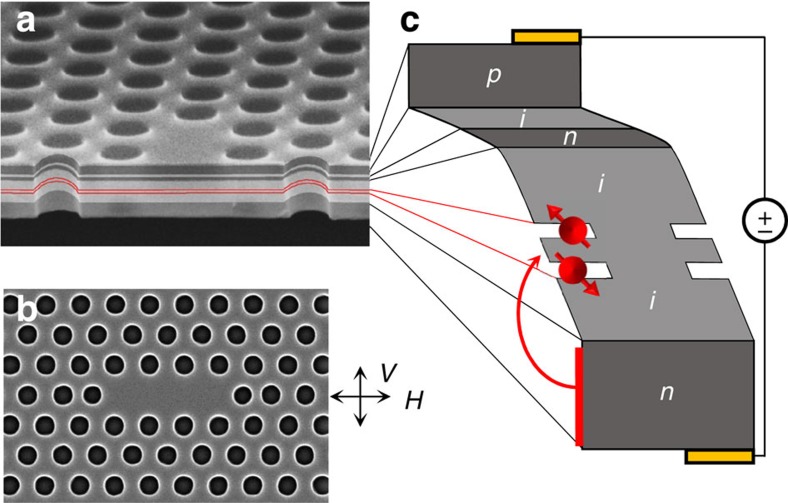
Cavity–QDM system. (**a**) Cross-sectional scanning electron microscopy (SEM) image of the doped GaAs photonic crystal membrane. Dark (light) grey regions correspond to doped (undoped) GaAs, while the thin red lines mark the position of the InAs QD layers. (**b**) Top-down SEM image of the GaAs photonic crystal L3 defect cavity. The vertical and horizontal polarization convention is defined in the inset as parallel (*V*) and orthogonal (*H*) to the cavity mode. (**c**) Band diagram of the doped photonic crystal membrane illustrating the two-electron QDM. Dark grey regions are *p* or *n-*type GaAs, while the light grey regions are undoped GaAs.

**Figure 2:**
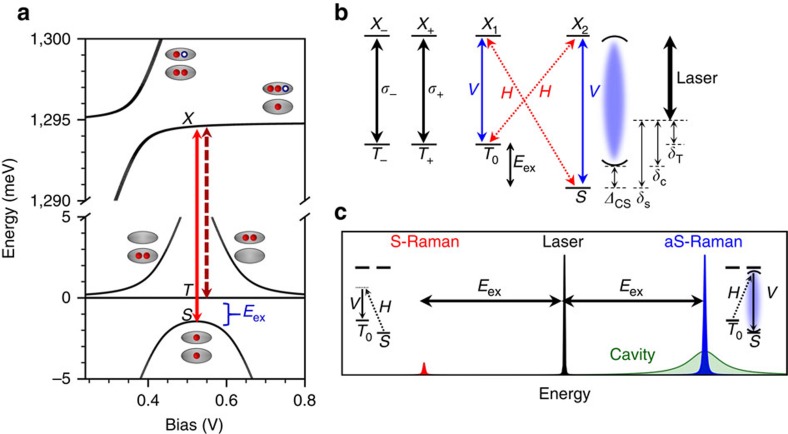
Cavity–QDM energy levels. (**a**) Energy levels of a QDM charged with two electrons as a function of applied bias. Both the excited and ground states are illustrated, the former in the upper part of the diagram. The singlet (*S*) and triplet (*T*) ground states are labelled as are the common excited states (*X*). Transitions to the singlet and triplet states are indicated by the solid and dashed arrows, respectively. (**b**) Level diagram illustrating the cavity–QDM system at a single bias. *V* (*H*) and the blue solid (red dashed) arrows indicate the polarization selection rules of the transitions as aligned (anti-aligned) with the vertical cavity mode polarization. The *T*_+_/*T*_−_ transitions are circularly polarized (black solid arrows). The cavity mode is represented by the pair of curved lines and the laser is the thick black arrow in the rightmost part of the diagram. The laser detunings from the singlet, triplet and cavity mode are defined as *δ*_S_, *δ*_T_ and *δ*_C_, respectively, and the cavity-singlet detuning is defined as *Δ*_CS_. (**c**) Schematic depicting the Stokes (red) and anti-Stokes (blue) Raman sidebands separated from the laser (black) by *E*_ex_. For this particular laser detuning, anti-Stokes Raman dominates because it is resonant with the cavity mode (green). Insets illustrate the optical processes that generate Raman photons.

The system consists of an InAs/GaAs QDM embedded within an L3 cavity of a GaAs photonic crystal membrane (Fig. 1a,b). In these studies we utilize the lowest-energy cavity mode, which has a polarization perpendicular to the long axis of the cavity. The photonic crystal membrane was doped during growth to form a diode that allows controlled loading of charge into the QDM and also tuning of the energy levels of the two QDs into resonance (Fig. 1c)^24^,^25^. The QDM structure was designed so that the energy levels are in resonance at a diode voltage for which two electrons are stable within the QDM^21^. Modelling this system and comparing to optical spectra (see Supplementary Fig. 1 and Supplementary Note 1) confirms that we obtain the two-electron system with the energy-level structure shown in Fig. 2a (refs 24, 25). The available ground states consist of a singlet (|*S*〉) and three degenerate triplets (|*T*_−_〉, |*T*_+_〉 and |*T*_0_〉)^24^. The singlet state is pushed down from the triplets because of electron tunnelling between the dots to give the kinetic exchange splitting (*E*_ex_). The magnitude of the exchange splitting is determined by the tunnelling rate, which depends on the width and height of the tunnel barrier, and on the effective mass of the carrier (that is, whether electron or hole)^21^,^25^. With a 9-nm GaAs barrier we obtain an electron tunnelling rate of 1.75 meV and an exchange energy of 1.45 meV.

The |*T*_0_〉 and |*S*〉 states couple to common excited states (|*X*_1_〉 and |*X*_2_〉), forming a double Λ-system qualitatively similar to the single QD trion^12^,^22^,^24^,^26^,^30^. This is illustrated in Fig. 2b along with the optical selection rules. As a result, spin–flip Raman transitions are possible, in which a laser drives population directly from one ground state to another with only a virtual population of the excited state. The spectral signature of Raman transitions is the appearance of sidebands shifted below and above the laser energy by the ground-state splitting, that is, the kinetic exchange splitting, *E*_ex_ (Fig. 2c). The lower-energy sideband is known as Stokes Raman emission and results in a population transfer from the singlet to the triplet (Fig. 2c left inset), while the higher-energy sideband is anti-Stokes Raman emission and transfers population from the triplet to the singlet (Fig. 2c right inset). Photons generated by the Raman process track the laser energy and have a linewidth determined by the spin-dephasing rate, not that of the optical transition. In contrast, transitions involving the |*T*_−_〉 and |*T*_+_〉 states do not participate in the Λ-configuration and behave as a pair of uncoupled two-level systems.

The large exchange energy splitting of the spin states allows each optical transition in the Λ-system to be separately brought into resonance with the cavity mode. The vertical polarization of the cavity mode couples to one leg of both Λ-systems. In the next part of this paper we align the polarization of the laser perpendicular to the cavity mode (horizontal polarization) and detect the emission with vertical polarization aligned parallel with the cavity mode: (laser, detection) polarization=(*H*,*V*). The cavity greatly enhances the emissive side of the Λ-system and stimulates the Raman process, while not enhancing the laser excitation field^6^,^32^. We illustrate this cavity-stimulated Raman process in Fig. 2c for the case where the higher-energy (anti-Stokes) Raman sideband emission is resonant with the cavity mode. Under this condition, the process is highly spin-selective in that the cavity only stimulates emission when starting from the |*T*_0_〉 state. In the final part of the paper we reverse the polarizations to (*V*, *H*) to enhance the excitation side and drive the Raman transitions into the nonlinear regime.

### Cavity-stimulated Raman emission

**Figure 3:**
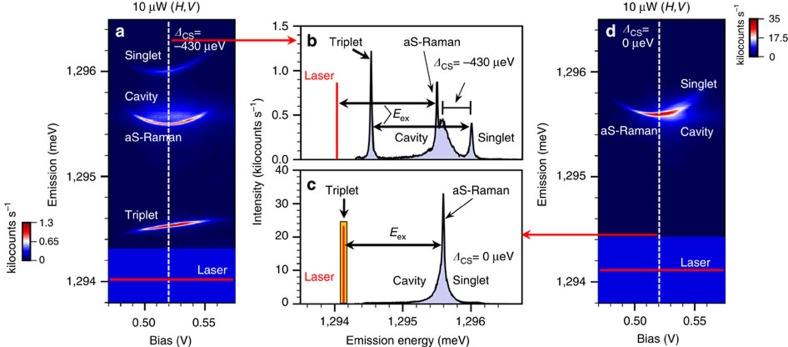
Bias-tunable QDM emission. (**a**) Quasi-resonant bias map of the emission spectra from the singlet, triplet and cavity mode at a detuning of *Δ*_CS_=−430 μeV (sample temperature of 5 K). Strong, bias-tunable anti-Stokes Raman emission is also observed. (**b**) Emission spectrum extracted from the dashed line in **a** at 520 mV. (**c**) Emission spectrum extracted from **d** at the dashed line (520 mV) for the case where the cavity is brought into resonance with the singlet transition (*Δ*_CS_=0 μeV) by raising the sample temperature to 22 K and where the laser is resonant with the triplet. Laser scatter prohibits observation of the triplet transition and so we indicate its known energy with the orange rectangle. (**d**) Resonant bias map for a cavity-singlet detuning of *Δ*_CS_=0 μeV. The polarization configuration is (laser, detection)=(*H*,*V*) for all spectra in this figure. The intensity in **a**,**d** is plotted on a linear scale in kilocounts s^−1^.

Optical transitions between the ground states (|*T*_0_〉 and |*S*〉) and the excited states have distinct spectral signatures in the bias-dependent emission spectra^20^,^24^,^25^. This is demonstrated in Fig. 3a where we excite the sample quasi-resonantly with the laser red-detuned from all transitions and with a polarization configuration of (laser, detection)=(*H*,*V*). Four spectral features are apparent in the bias map in Fig. 3a. These spectral features are observed over a bias range of 0.49–0.56 V, corresponding to the stability range of the two-electron charge state. The spectrum for a fixed bias near the centre of this range is shown in Fig. 3b. The narrow features at ∼1,294.5 and ∼1,296 meV correspond to photoluminescence (PL) from the triplet and singlet transitions, respectively, while the broader feature at ∼1,295.5 meV is because of emission from the cavity. These emission features presumably arise from phonon-assisted excitation of the system since the laser is detuned. The fourth spectral line at ∼1,295.5 meV in Fig. 3a,b originates from an anti-Stokes Raman process (Fig. 2c, right inset). This line appears 1.45 meV above the laser energy, a value equal to *E*_ex_, and is significantly sharper in linewidth than the other transitions. To clearly observe each of the lines in these spectra, the Raman emission was slightly detuned from the cavity, and also the cavity was detuned from the singlet transition (*Δ*_CS_=*E*_C_–*E*_S_=−430 μeV). In Fig. 3c,d, the temperature was increased to redshift the singlet into resonance with the cavity (*Δ*_CS_=0 μeV), and the laser shifted up to the triplet transition (*δ*_T_=*E*_L_–*E*_T_=0 μeV) to bring the Raman line into resonance with both. Under this doubly resonant condition, the anti-Stokes Raman emission dominates the spectrum and is more than an order of magnitude stronger than the detuned case in Fig. 3b. We note that the presence of Raman emission over the entire stability range under resonant excitation implies that optical pumping does not occur. This is because of the large exchange energy that makes spin relaxation via co-tunnelling to the *n-*type contact too fast for optical pumping^12^.

The cavity linewidth (*κ*) is ∼190 μeV (*Q*∼6,800) and the singlet and triplet linewidths in Fig. 3b are 40 and 25 μeV, respectively, with a cavity-singlet detuning of *Δ*_CS_=−430 μeV. The QDM linewidths are broadened through coupling to the cavity and thus depend on cavity-detuning. A cavity–QDM coupling constant *g*≈57 μeV was found by measuring the anticrossing when the cavity line was tuned through the triplet PL line via a gas adsorption technique (see Supplementary Fig. 2 and Supplementary Note 2)^33^. The anti-Stokes Raman linewidth in Fig. 3b is resolution-limited; however, using a scanning Fabry–Perot interferometer, the Raman linewidth was measured to be ∼11 μeV at the centre of the two-electron bias range, which is where the sensitivity to electric field fluctuations is minimal. We tentatively attribute this linewidth to the fast spin relaxation because of co-tunnelling. It should be possible to greatly decrease this co-tunnelling rate by optimizing the layer thicknesses in the structure. When the bias was increased or decreased away from the central bias, the Raman linewidth increased in a way expected for electric field fluctuations (see Supplementary Fig. 2, Supplementary Note 2, Supplementary equation 1). Power dependence of the Raman emission is discussed in Supplementary Fig. 4 and Supplementary Note 4.

**Figure 4:**
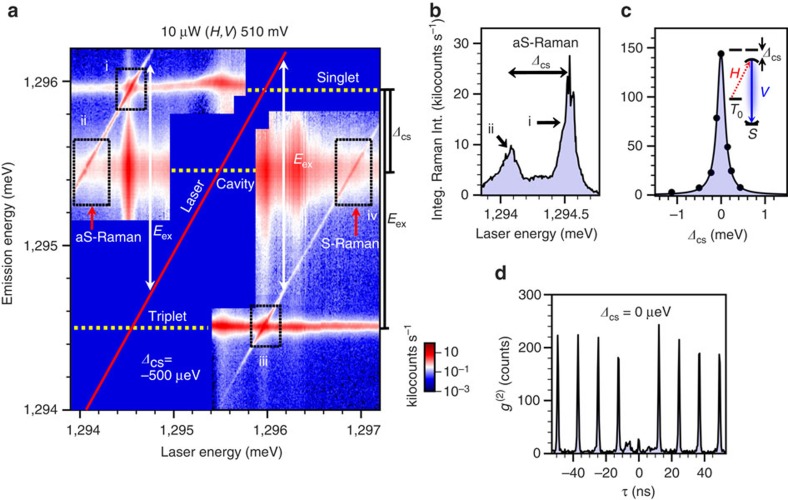
Cavity-stimulated Raman emission. (**a**) Two-dimensional spectral map of the emission intensity as a function of laser and emission energy. The intensity is plotted on a logarithmic scale in kilocounts s^−1^. Non-shifting features correspond to PL from the singlet, triplet and cavity. A pair of narrow emission lines track the laser energy and are separated from the laser by *E*_ex_. These features are identified as anti-Stokes and Stokes Raman. Excitation power, polarization configuration and bias are indicated at the top of the figure. The labelled boxes identify resonant and cavity-stimulated Raman for the anti-Stokes (i, ii) and Stokes (iii, iv) Raman. (**b**) Integrated intensity of the anti-Stokes Raman emission from **a** versus laser energy at a fixed bias of 510 mV. The laser power is above saturation for resonant excitation conditions. Resonant and cavity-stimulated Raman peaks correspond to Boxes i and ii in **a**, respectively. (**c**) Integrated intensity of the cavity-stimulated anti-Stokes Raman versus *Δ*_CS_ at a fixed bias of 510 mV. The laser power was set to 10 μW, which is below saturation for all values of *Δ*_CS_. The data fit a Lorentzian function with a full-width at half-maximum of 212 μeV, similar to the cavity linewidth of 190 μeV. (**d**) Intensity autocorrelation measurement using pulsed laser excitation of the anti-Stokes Raman in a near-resonant condition. The sample bias was at 510 mV and the polarization configuration is (*H*,*V*). The cavity-singlet detuning is *Δ*_CS_=0 μeV to maximize Raman intensity and the laser-detuning was chosen to minimize the cavity background. We obtain *g*^(2)^(0)≈0.14, a clear sign of antibunching.

We show in more detail the emission spectrum as a function of laser detuning in Fig. 4a with a two-dimensional spectral map. Here we plot the emission intensity as a function of both emission and laser energy for fixed bias (510 mV) and cavity-singlet detuning (*Δ*_CS_=−500 μeV). The laser scatter is blocked by a polarizer and the spectrometer, and only its energy is indicated by the red line. Both the Stokes and anti-Stokes Raman lines are observed as diagonal lines offset from the laser by ±*E*_ex_. When the laser crosses the singlet, triplet and cavity lines, indicated by dashed lines, there are strong resonant enhancements in each of the emission intensities. (Note that an additional enhancement in the cavity emission at a laser energy of ∼1,296.3 meV arises from a weak transition in the one-electron charge state not visible in the spectral map.) As the laser is scanned across the triplet there is a large enhancement in the anti-Stokes Raman intensity (Box i in Fig. 4a) that corresponds to a resonant Raman process, which drives the system into the singlet spin state. Decreasing the laser energy further brings the anti-Stokes Raman into resonance with the cavity, resulting in a broad enhancement due to the cavity-stimulated Raman process (Box ii in Fig. 4a). The Stokes Raman emission behaves in a similar manner but drives the system into the triplet spin state. The cavity emission peaks under resonant excitation of either the singlet or the triplet due to well-established non-resonant cavity-feeding mechanisms.

Although Raman is usually only significant under near-resonant excitation, a cavity greatly enhances the Raman process leading to emission over a much wider range. We illustrate this by extracting the anti-Stokes Raman intensity from Fig. 4a as a function of laser energy to give the Raman excitation spectrum (Fig. 4b). These intensities were obtained by fitting each spectrum to a sum of three Lortenzians in order to account for the cavity, singlet and anti-Stokes Raman emission features. The Raman excitation spectrum was acquired at 10 μW (above saturation for the resonant Raman process) and clearly shows Raman emission over an ∼700 μeV range with peaks occurring when the Raman emission is resonant with the singlet and the cavity. Note that the intensities at these two resonances are comparable, suggesting that cavity-stimulated Raman can occur with high count rates and over a wide range. The cavity-stimulated Raman process is still highly spin-selective even with the laser detuned below the triplet by 0.5 meV; that is, the anti-Stokes Raman spin–flip process is much stronger than the Stokes. The Stokes emission under these conditions could be obtained by following the Stokes emission line in Fig. 4a down to a laser energy of 1,294 meV. Stokes emission is far too weak to measure at that point; however, extrapolating a fit to the Stokes emission decay with detuning gives a value 1,000 times weaker than the measured anti-Stokes value. This large tuning range combined with the spin selectivity of the Raman process is important for proposed scalable quantum networks^34^, where a spin qubit must transfer its state to a photon that may need to be tunable in order to interact with other spin qubits that have optical transitions with dissimilar energies^6^. As the cavity-detuning *Δ*_CS_ is decreased by temperature tuning, the cavity-stimulated anti-Stokes Raman intensity increases further, as shown in Fig. 4c, with a resonance linewidth of 212 μeV determined by the cavity linewidth.

To test the single-photon nature of the Raman emission, we have measured the second-order intensity autocorrelation function, *g*^(2)^(*τ*), at *Δ*_CS_=0 μeV (Fig. 4d). The excitation power was kept low (240 nW) to reduce the effects of laser scatter and background cavity emission. We find that the Raman photons are antibunched with a two-photon probability of *g*^(2)^(0)=0.14. We attribute the non-zero two-photon probability to laser background and possible feeding of the cavity mode by other optical processes. The purity of this two-photon emission can be improved by introducing a bandpass with an appropriate Fabry–Perot filter. We note that the absence of optical pumping in this sample is an advantage for a single-photon source because the system reinitializes itself and therefore does not require a separate initialization pulse^35^.

### Strongly driven QDM

**Figure 5:**
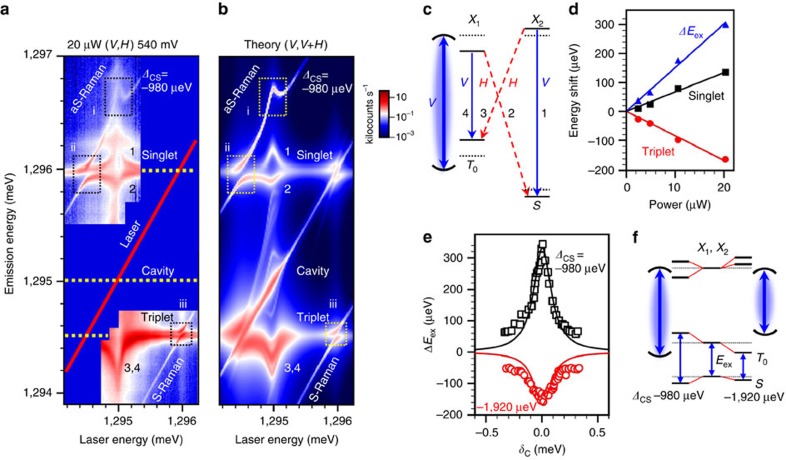
AC Stark shifts of a two-spin system. (**a**) Two-dimensional spectral map taken in a (*V*, *H*) polarization configuration demonstrating cavity-enhanced AC Stark shifts at *Δ*_CS_=−980 μeV. The intensity is plotted on a logarithmic scale in kilocounts s^−1^ and the numbered features correspond to the transitions in **c**. The labelled boxes identify the Stark-shifted anti-Stokes Raman (i) and the Autler–Townes splittings (ii, iii). (**b**) Theoretical spectral map calculated from the experimental values in **a**. Both vertically polarized and horizontally polarized emission components (*V*,*V*+*H*) are required to reproduce the experimental data. The Mollow triplet structure near the laser line is observed in the simulation but not resolved in our experiments because of the substantial laser scatter. (**c**) Energy-level diagram illustrating the AC Stark shifts induced by the driven cavity. Solid (dashed) horizontal lines denote the modified (original) state energies. Blue solid arrows represent excitation and emission processes with the cavity polarization (*V*), while the red dashed arrows indicate *H*-polarized emission. The curved lines represent the cavity mode at the detuning in **a**. (**d**) Power dependence of the AC Stark shifts on the singlet (black squares) and triplet (red circles) levels. We obtain the shift in *E*_ex_ (blue triangles) by taking the difference of the singlet and triplet level shifts. (**e**) AC Stark shift of the exchange energy (*ΔE*_ex_) extracted from **a** (black squares, *Δ*_CS_=−980 μeV) and at a different cavity energy (red circles, *Δ*_CS_=−1920 μeV) versus the laser-cavity-detuning, *δ*_C_. Lortenzian curves with the 190 μeV cavity linewidth are included as guides to the eye. (**f**) Level diagram illustrating how the sign of *ΔE*_ex_ depends on the cavity detuning.

Thus far, we have demonstrated cavity-stimulated Raman emission using a polarization configuration such that the cavity enhances the emission process, but not the laser field. We now show how the spectra change when driven strongly by the laser field. This is performed by inverting the polarization configuration such that the laser polarization is now parallel to the cavity (*V*, *H*). The spectral map of the emission as a function of laser energy is shown in Fig. 5a. In contrast to the prior data in Fig. 4a, we now see the development of very large Autler–Townes splittings and AC Stark shifts both in the PL and Raman lines. In our case, the use of an engineered two-spin Λ-system allows us to realize the effects of strong driving on the singlet–triplet ground states via Raman transitions and to open a novel path towards spin qubit control.

The spectra are complex but can readily be understood in terms of the cavity-enhanced Autler–Townes effect. If the drive field is made sufficiently strong, any two levels resonant with the laser field become ‘dressed' and form polariton-like states. As a result, both the ground and excited states split, forming pairs of levels split by the generalized Rabi frequency 
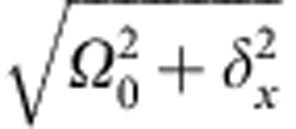
, where *Ω*_0_ is the Rabi frequency and *δ*_*x*_ with *x*=S, T is the laser detuning from the transition (singlet or triplet). At resonance where *δ*_*x*_=0 the Autler–Townes splitting is given by *Ω*_0_. It can be resolved directly in a three-level system by observing emission between the dressed state and a spectator state. In the limit of large detuning (*Ω*_0_/*δ*_*x*_≪1) the eigenstates are only weakly dressed and experience AC Stark shifts, *Ω*_0_^2^/4*δ*_*x*_, which scale linearly with the laser intensity, in contrast to the Autler–Townes splitting, which goes as the square root (see Supplementary Note 5).

The cavity is strongly driven by the co-polarized laser, and, as a result, a steady-state electric field is generated. Its effect is felt by the QDM as an additional driving field, so that *Ω*_0_ is replaced by the cavity-enhanced Rabi frequency *Ω* (see Supplementary Note 6):


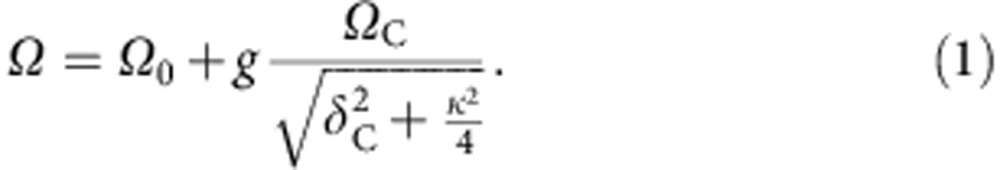


Here *g* is the coupling of the QDM to the cavity, *Ω*_C_ is the coupling of the laser to the cavity, *δ*_C_ is the detuning of the laser from the cavity (Fig. 2b) and *κ* is the cavity linewidth. The role of the cavity is twofold; it enhances the effective laser power that drives the QDM and it also causes an additional detuning dependence of the spectra through the term in the denominator of equation (1). We discuss these effects for the AC Stark shifts and the Autler–Townes splittings, first for relatively large detuning as in Fig. 5, and then for the cavity-resonant with a QDM transition.

In Fig. 5a,b there is a dramatic AC Stark shift effect of all spectral features when the laser crosses the cavity. The cavity is spectrally between the triplet and singlet transitions, red-detuned from the singlet by 980 μeV. The laser dresses the states even though it is well detuned from the QDM transitions because of the cavity-induced field enhancement^36^. As illustrated in the level diagram of Fig. 5c, the AC Stark shift of transitions 1 and 4, which are aligned with the cavity polarization, results in shifts of all four transitions. For the present cavity-singlet detuning, the singlet transitions 1 and 2 shift to the blue and red, respectively, whereas the triplet transitions 3 and 4 both redshift.

We note that, while the agreement between the experimental (Fig. 5a) and theoretical (Fig. 5b) emission lines and their energy shifts is excellent, there are significant variations in the measured emission intensity and linewidth not captured by our model. In particular, we should only observe the horizontally polarized transitions 2 and 3 in the (*V*, *H*) configuration (see Supplementary Fig. 5 and Supplementary Note 7). However, because of imperfect polarization rejection in the experiment, there is leak-through from the vertically polarized transitions that is greatly enhanced by the cavity. In fact, all four PL transitions appear with comparable intensity in the experiment. To account for these ‘forbidden' lines we include both *H* and *V* spectra in our calculations. Because the theory accounts only for cavity-enhanced excitation, not emission (Supplementary Note 6), the *V* spectra are included with equal weight compared with the *H* spectra to better match experiment. Even so, there are still discrepancies in the relative intensities between experiment and theory that may be due to non-resonant excitation processes (for example, phonon-assisted absorption) not included in the theory. Moreover, the QDM lines broaden and the integrated intensity decreases substantially when the laser is swept over the cavity. These effects may arise from phonon interactions that have recently been shown to have a significant effect on linewidths in strongly driven two-level systems^13^,^14^,^15^,^16^,^17^.

In contrast to the two-level systems, we also observe the effect of the AC Stark shift on the ground-state singlet–triplet splitting. This manifests most dramatically as an AC Stark shift of the anti-Stokes Raman emission (Box i, Fig. 5a) and represents a modulation of the spin interaction by cavity photons. When the cavity is between the singlet and triplet transitions, as in Fig. 5a,b, the exchange energy, *E*_ex_, increases because of the AC Stark effect. The dependence of *E*_ex_ on cavity-laser detuning obtained from these data is plotted as black squares in Fig. 5e. Alternatively, *E*_ex_ can be decreased by tuning the cavity to the red or blue of both transitions. This is shown for a red cavity-singlet detuning of *Δ*_CS_=−1,920 μeV as red circles in Fig. 5e (see Supplementary Fig. 6 and Supplementary Note 8). The level diagrams for these two cases are illustrated in Fig. 5f. As expected, we find a linear power dependence for the measured AC Stark shift of the singlet and triplet levels, and therefore for the exchange energy (Fig. 5d). This effect is highly promising for advancing scalable quantum networks as it provides a local, all-optical method to tune spin interactions in a single quantum emitter.

**Figure 6:**
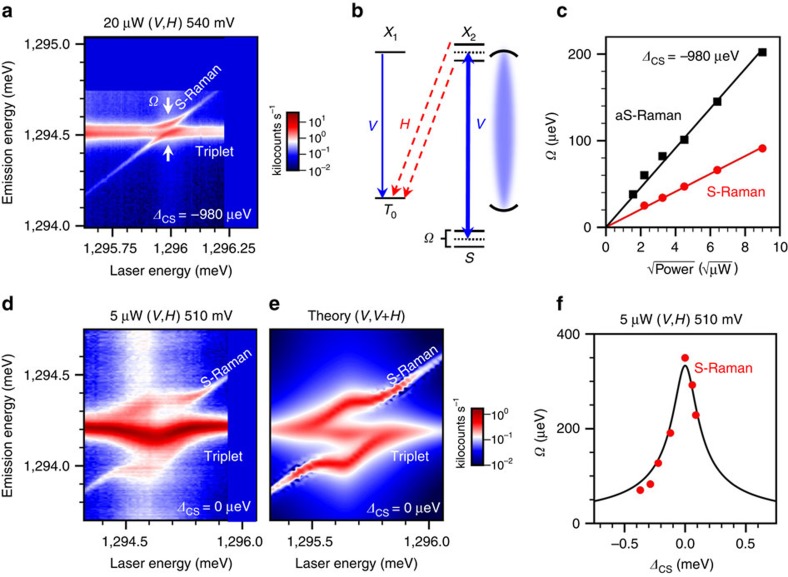
Cavity-enhanced Rabi frequency. (**a**) Magnification of the Stokes Raman Autler–Townes splitting in Fig. 5a under 20 μW excitation and with *Δ*_CS_=−980 μeV. The intensity is plotted on a logarithmic scale in kilocounts s^−1^. (**b**) Energy-level diagram illustrating the origin of the Autler–Townes splitting. Solid (dashed) horizontal lines denote the modified (original) state energies. Blue solid arrows represent excitation and emission processes with the cavity polarization (*V*), while the red dashed arrows indicate *H*-polarized emission. The curved lines represent the cavity mode at the detuning in **a**. (**c**) Power dependence of the Autler–Townes splitting for the anti-Stokes (black squares) and Stokes (red circles) Raman when *Δ*_CS_=−980 μeV. Solid lines are fits to the data. (**d**) Stokes Raman Autler–Townes splitting emission for *Δ*_CS_=0 μeV and under 5 μW excitation. The intensity is plotted on a logarithmic scale in kilocounts s^−1^. The anticrossing displays a bowing effect that is due to the enhancement of the Rabi frequency as the laser is scanned over the cavity. (**e**) Theoretical spectral map calculated from the experimental values in **d**. Both vertically polarized and horizontally polarized emission components (*V*, *V*+*H*) are required to reproduce the experimental data. (**f**) Stokes Raman Autler–Townes splitting (*Ω*) versus *Δ*_CS_ under 5 μW excitation. The data were fit to equation (1) where *κ*=190 μeV, *Ω*_0_=5.6 μeV and *Ω*_C_=546 μeV. We choose *Ω*_C_/*Ω*_0_=97.5 in agreement with our simulations, and thus *Ω*_C_ is the only free parameter.

Large Autler–Townes splittings in the Raman lines are observed when the laser crosses the triplet or singlet transitions (Boxes ii and iii in Fig. 5a,b). A magnified view of the data in Box iii (Stokes Raman) is shown in Fig. 6a for the case of large cavity-detuning (*Δ*_CS_=−980 μeV). The corresponding level diagram is shown in Fig. 6b. The magnitude of the anticrossings grows as the square root of the laser power for both the Stokes and anti-Stokes Raman (Fig. 6c), as expected. The asymmetry in magnitude between the Stokes and anti-Stokes Raman arises from the cavity-laser detuning *δ*_C_ appearing in equation (1).

The enhancement of the Rabi frequency becomes extreme when the singlet is redshifted into resonance with the cavity as seen in Fig. 6d,e. We observe a large Autler–Townes splitting in the Stokes Raman emission with a Rabi frequency that shows a strong resonance profile as the laser is tuned across the singlet-cavity double resonance. The PL line going through the anticrossing in Fig. 6d is believed to arise from the vertical triplet transition as well as the *T*_+_/*T*_−_ transitions and is discussed further in the Methods. With a magnitude of 350 μeV, the cavity-resonant Autler–Townes splitting is ∼35 times larger than that achieved in a QDM outside of a cavity^12^. As shown in Fig. 6f, the full resonance profile of the Autler–Townes splitting as a function of cavity-singlet detuning fits equation (1) well, where we substitute −*Δ*_CS_ for *δ*_C_ to fix the laser to the singlet transition. This result is essentially a demonstration of cavity QED in the limit where the number of cavity photons is large, moving well up the Jaynes–Cummings ladder. The bare cavity–QDM coupling is *g*≈57 μeV, but in the presence of *n* cavity photons the interaction is 
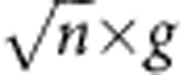
. For a 350-μeV splitting, this indicates the presence of ∼10 cavity photons. Aside from the interesting physics of this system, this large splitting represents a very strong light–matter interaction that may result in even faster optical control of this quantum system.

## Discussion

We have demonstrated an engineered quantum system where the spin states, optical transitions, charge state and photonic environment can all be carefully controlled through design. This enables us to realize the first two-spin–cavity system and subsequently explore the cavity-stimulated Raman process as well as cavity-enhanced nonlinearities in the limit that the ground-state splitting is much larger than the cavity linewidth. One natural application of the cavity-stimulated Raman process is as a tunable, indistinguishable, single-photon source^37^,^38^. This is because the coherent Raman process can produce single photons with the same temporal lineshape as the laser^39^, which for short pulses can far exceed any spectral wandering^6^,^40^. However, we anticipate that the impact of the cavity–QDM system will be far broader. In particular, this system could be used as a node in a quantum network, where the spin degree of freedom would act as a stationary qubit that can be entangled with a photon for long-distance interactions with other nodes. The Raman process is highly spin-selective, allowing for the conversion of the spin state to a photon number state. While the spin lifetime in this particular sample is designed to be short, the spin coherence time can be made much longer (superior to a single-electron spin) by engineering the QDM structure to be insensitive to fluctuating electric and magnetic fields^30^ or by utilizing hole spins^28^,^41^,^42^. This system also allows for tuning of both the emitted photon energy and the exchange energy. The cavity-stimulated Raman emission can be tuned by shifting the laser energy in conjunction with the cavity resonance, and the spin exchange energy can be optically controlled through the cavity-enhanced nonlinearities. Importantly, these capabilities are local, providing a means to tune photons and spin interactions in spatially separated nodes of a quantum network. Finally, the AC Stark effect utilized here can be implemented on ultrafast timescales^43^, which may open new avenues for quantum information processing^44^. Combining the design potential of photonic crystal architectures with the heterostructure and doping capability of semiconductor nanostructures creates a versatile platform for solid-state quantum technology.

## Methods

### Sample

InAs QDMs were grown by molecular beam epitaxy within a GaAs diode on an 800-nm Al_0.7_Ga_0.3_As sacrificial layer. The diode doping was *n-i-n-i-p* with layer thicknesses of 50, 95, 10, 10 and 30 nm. This heterostructure enabled charging of the QDMs at low currents, both preventing deleterious heating effects and improving overall device performance. The QDs are located 40 nm above the lower *n*-type layer and are separated by a 9-nm GaAs tunnel barrier. The QD closest to the back *n*-type layer was 2.5 nm in height, while the top QD was 3 nm and therefore lower in energy by ∼30 meV. It is this lower-energy dot that is resonantly excited. The dots are distributed randomly in the growth plane with a density of several QDMs per μm^2^.

The two-dimensional photonic crystal consisted of a triangular lattice of holes (62 nm radius) with a lattice constant of 244 nm that were etched through the diode epilayer into the AlGaAs sacrificial layer. Three missing holes at the centre formed an L3 cavity (Fig. 1b), and we use the lowest-energy cavity mode for our studies. The polarization of this mode is orthogonal to the long axis of the cavity. The two end holes of the cavity were shifted by 17% of the lattice constant and were also 2 nm smaller in radius^45^. This pattern was defined by electron-beam lithography and a Cl_2_-based inductively coupled plasma etch. The AlGaAs was removed under each photonic crystal using a hydrofluoric acid (HF) wet-etch, leaving behind a free-standing 195-nm-thick photonic crystal membrane. Ohmic contacts to the diode are made with indium, enabling charge control of the QDMs.

### Measurement

All measurements were carried out in a closed-cycle helium cryostat using a confocal microscope. We excite the sample with tunable diode laser that passes through a polarizing beamsplitter and an achromatic half-wave plate to orient its polarization relative to the cavity mode. Emission is collected in a cross-polarized configuration to reject laser scatter and is directed to a triple spectrometer in additive mode with a resolution of ∼15 μeV. This gives a good combination of stray light rejection, spectral resolution, and multi-spectral detection. A background spectrum is collected and subtracted at each laser energy to further reduce scatter.

In the *g*^(2)^(*τ*) measurements a pulsed laser (∼25 ps full-width at half-maximum, 80 MHz repetition rate) excites the sample and the collected emission is filtered by a single spectrometer with an ∼90 μeV bandpass window. The Raman photons are then directed into a Hanbury Brown–Twiss interferometer where they are detected by a pair of single-photon counting modules connected to an autocorrelator board. The laser power is kept low (240 nW) to reduce the contribution of cavity emission from other optical processes.

### Theory

Our theoretical calculations are intended to describe the effective cavity-induced driving of the QDM (see Supplementary Note 6). The Hamiltonian of the system is *H*=*H*_0_+*H*_*QD–L*_+*H*_*QD–C*_+*H*_*C–L*_, where the terms, respectively, describe the interaction-free cavity–QDM system, the QDM–laser interactions, the coupling between the QDM and the cavity, and finally the interaction between the cavity and the laser. Relaxation is accounted for via the standard Lindblad operators and we include a pure dephasing term for the optical transitions. We subsequently solve for the QDM emission spectra by taking the Fourier transform of the two-time correlation function.

In modelling the QDM system we assumed that the *T*_+_/*T*_−_ transitions from the |*T*_−_〉=|↓↓〉 and |*T*_+_〉=|↑↑〉 spin states are not part of the Λ-configuration (Fig. 2b) and behave as a pair of uncoupled two-level systems. This differs from what has been done in previous work where it was assumed that the three triplets were mixed and could be treated as a single state^12^,^26^. Here we account for them separately because their transitions have different polarization selection rules and are therefore dressed by the laser differently than the |*T*_0_〉 and |*S*〉 transitions in the strongly driven experiments.

## Additional information

**How to cite this article:** Vora, P. M. *et al*. Spin–cavity interactions between a quantum dot molecule and a photonic crystal cavity. *Nat. Commun.* 6:7665 doi: 10.1038/ncomms8665 (2015).

## Supplementary Material

Supplementary Information Supplementary Figures 1-6, Supplementary Notes 1-8 and Supplementary References
