# Supplementary material for: Spin–cavity interactions between a quantum dot molecule and a photonic crystal cavity
Source: Nat Commun. 2015 Jul 17;6:7665. doi: 10.1038/ncomms8665 (PMC4518300; doi:10.1038/ncomms8665)
Supplement: Supplementary Information — Supplementary Figures 1-6, Supplementary Notes 1-8 and Supplementary References [file ncomms8665-s1.pdf]

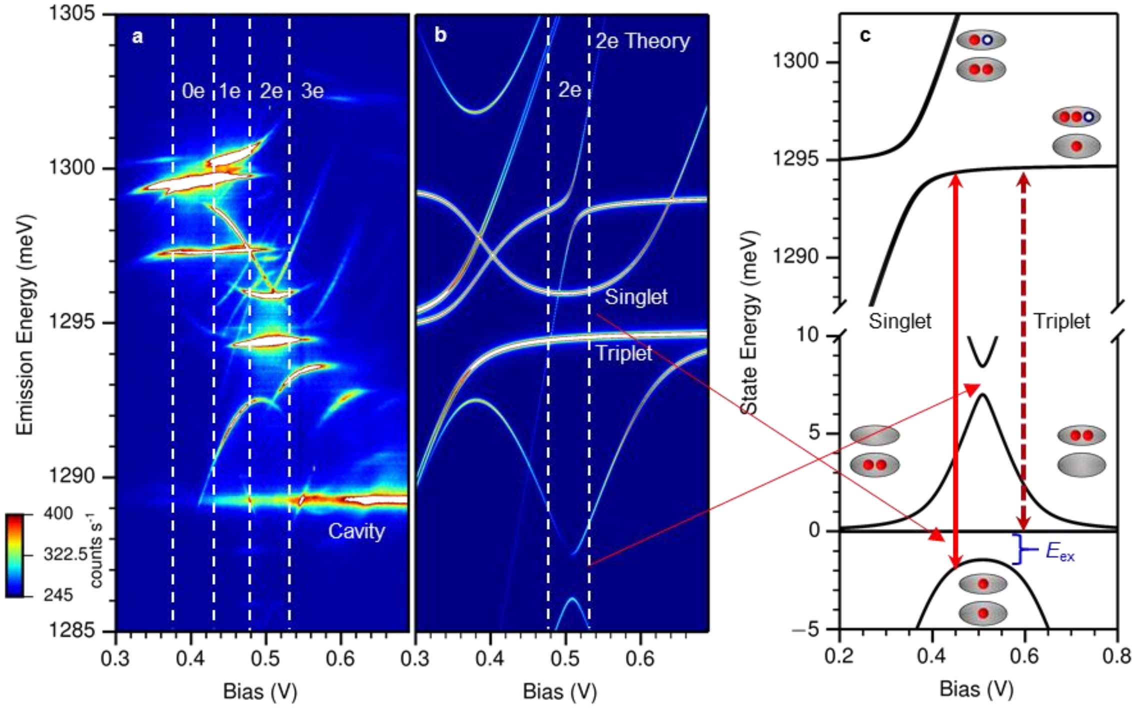

Supplementary Figure 1 | Level structure of a doubly charged QDM (a) PL bias map acquired under 90 nW non-resonant excitation at 860 nm. Charging steps are labeled by the vertical dashed lines. Intensity is plotted on a linear scale in counts s^{-1} and has been allowed to saturate to bring out weak features. (b) Transition spectrum calculation of the two-electron system. Model parameters are derived from the data in (a). (c) Energy levels of a QDM charged with two electrons as a function of applied bias used to calculate the transition spectrum in (b).

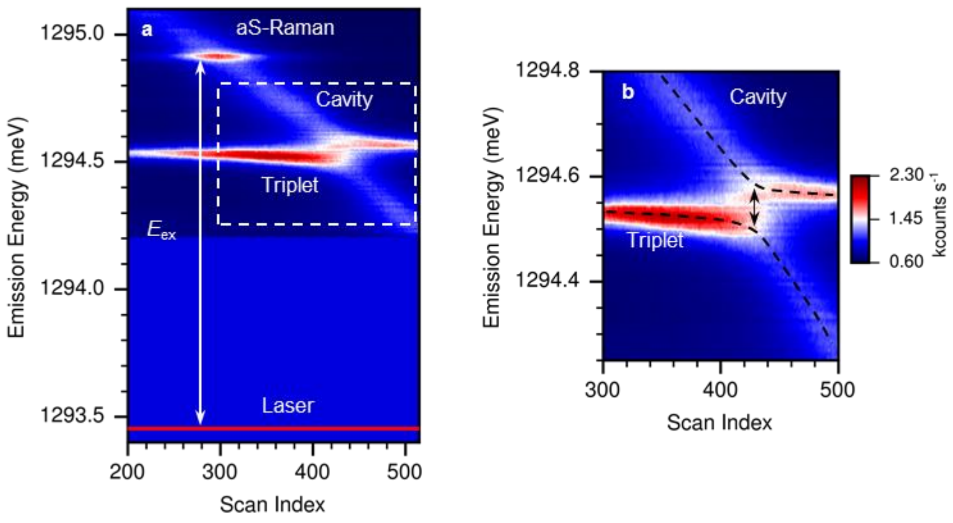

Supplementary Figure 2 | Cavity-QDM coupling measurement (a) The cavity was shifted through the aS-Raman as well as the triplet transition by a gas adsorption technique. Intensity is plotted on a linear scale in kilocounts s^{-1}. (b) Magnified view of the cavity-triplet anticrossing where the dashed black lines are guides to the eye.

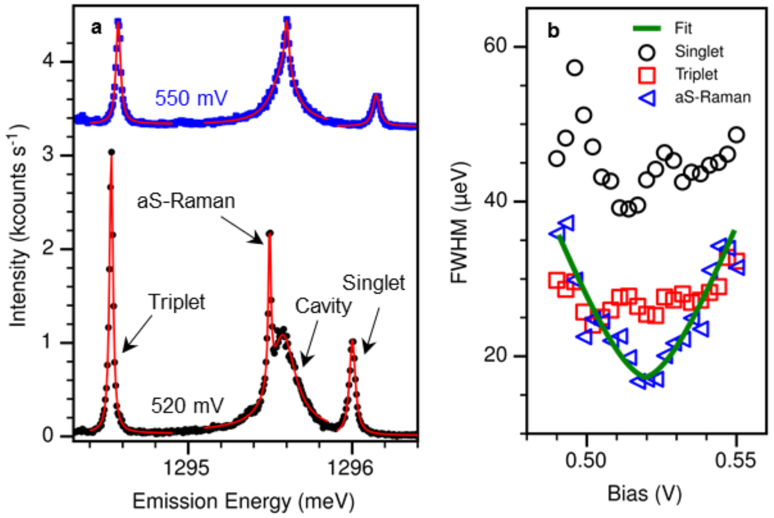

Supplementary Figure 3 | Bias dependence of the Raman linewidth (a) PL scans extracted from Fig. 3a at 520 mV (black circles) and 550 mV (blue squares). Example one and two Lorentzian fits to the data are shown as solid red lines and were used to extract the FWHM linewidth of the transitions. (b) FWHM linewidth of the singlet (black circles), triplet (red squares), and anti-Stokes Raman (blue triangles) as a function of bias extracted from the data in Fig. 3a. The solid green line is a fit of the aS-Raman linewidth to the model described in the text.

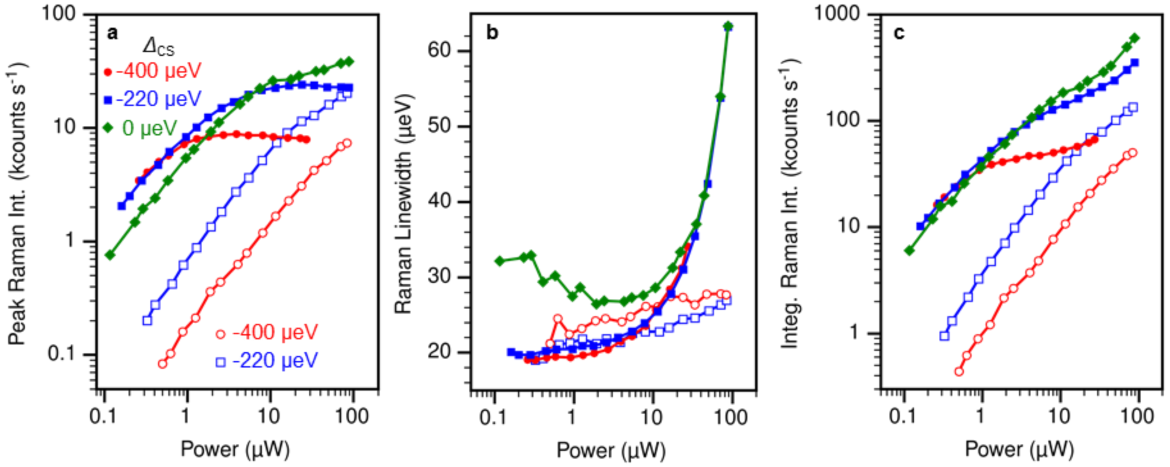

Supplementary Figure 4 | Power dependence of the Raman process (a) Power dependence of the anti-Stokes Raman peak intensity when the emission is resonant with the singlet (solid markers) and the cavity (open markers) at 510 mV for three detunings. (b) Power dependence of the anti-Stokes Raman linewidth for the same experimental parameters as in (a). (c) Integrated intensity of the anti-Stokes Raman versus laser power for the same experimental parameters as in (a). All figures share the legend in (a).

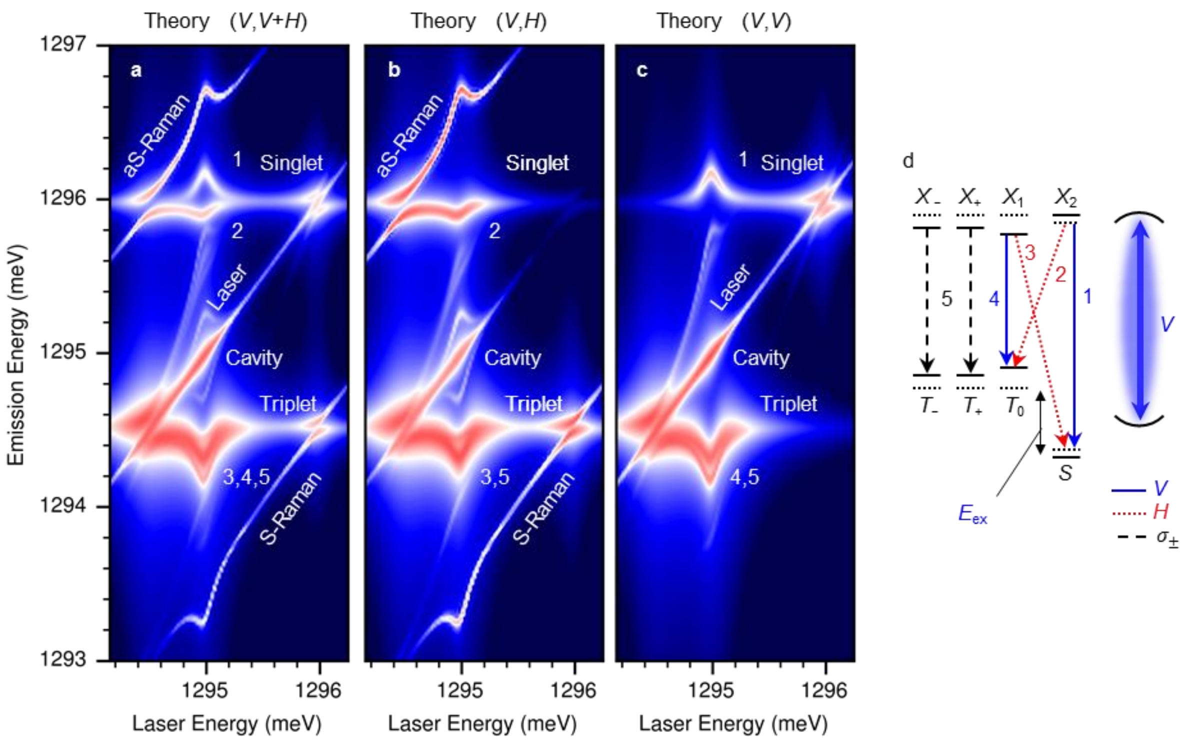

Supplementary Figure 5 | Polarization dependence of the calculated cavity-QDM spectra Two-dimensional spectral maps calculated from the experimental parameters in Fig. 5a where Δ_{CS} = -980 μeV and the laser is co-polarized with the cavity mode. (a) Map including both *V* and *H* emission with equal magnitudes. (b) Pure *H* emission map, corresponding to recombination via diagonal transitions 2 and 3. Additional contributions from T_+/T_- (transition 5) are also present. (c) Pure *V* emission map, corresponding to recombination via vertical transitions 1 and 4. Additional contributions from T_+/T_- (transition 5) are also present. (d) Level diagram illustrating the unique AC Stark shifts experienced by each transition. Polarization selection rules are labeled and defined in the legend. Solid (dashed) horizontal lines denote the modified (original) state energies. The blue solid arrows represent excitation and emission processes with the cavity polarization (*V*) while the red dashed arrows indicate *H* polarized emission. The curved lines represent the cavity mode at the detuning in (a).

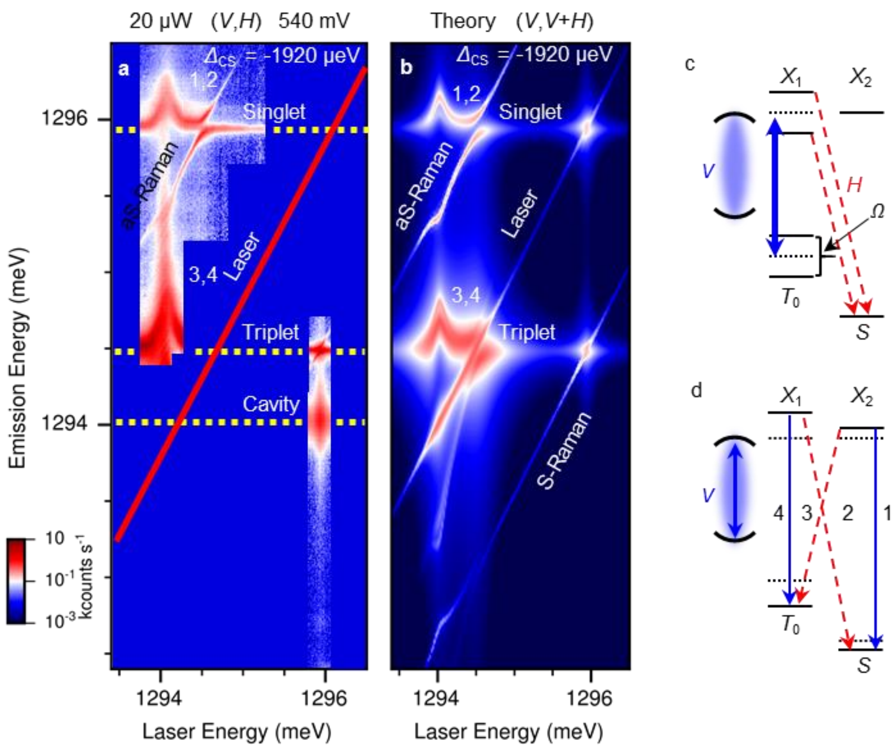

Supplementary Figure 6 | Control of the spin exchange energy (a) Two-dimensional spectral map taken in a (*V*, *H*) polarization configuration demonstrating cavity-enhanced AC Stark shifts and Autler Townes splittings at Δ_{CS} = -1920 μeV. The intensity is plotted on a logarithmic scale in kilocounts s^{-1} and the numbered features correspond to the transitions in (d). (b) Theoretical spectral map calculated from the experimental values in (a). Both vertically polarized and horizontally polarized emission components (*V*,*V*+*H*) are required to reproduce the experimental data. The Mollow triplet structure near the laser line is observed in the simulation but not resolved in our experiments due to the substantial laser scatter. (c,d) Energy level diagrams illustrating the origin of the (c) Autler-Townes splitting and (d) the AC Stark shifts. Solid (dashed) horizontal lines denote the modified (original) state energies. The blue solid arrows represent excitation and emission processes with the cavity polarization (*V*) while the red dashed arrows indicate *H* polarized emission. The curved lines represent the cavity mode at the detuning in (a).

Supplementary Note 1. QDM energy level structure

Charged QDMs exhibit bias-dependent photoluminescence spectra that are considerably more complicated than single QDs, but are well understood.[1] This is apparent in Supplementary Fig. 1a, where we present a PL bias map obtained under nonresonant excitation with a Ti-sapphire laser at 860 nm. In this particular set of data we have red detuned the cavity by gas adsorption in order to prevent the bright cavity emission from obscuring spectral features. Charging steps are observed which we assign to the 0e, 1e, 2e, and 3e charge states. Direct calculations of the doubly charged QDM transition spectrum following the methods of Refs. 1 and 2 using a tunnel coupling $t \sim 1.75$ meV agree with the experiment over the range where the two electron state is stable, reproducing the singlet, triplet, and other weak transitions in the data (Supplementary Fig. 1b). Additional features such as the "x-patterns" common in QDM spectra appear in the calculation but are absent from experiment due to the fact that they lie outside the bias region where the two electron state is stable. In Supplementary Fig. 1c we plot the energies of the singlet, triplet, and the excited states in a doubly charged QDM from which the spectrum in Supplementary Fig. 1b can be obtained by taking the difference between the excited and ground states to obtain the transition energies and then using the state vectors to determine the intensity.

Supplementary Note 2. Cavity-QDM coupling

We extract the cavity-QDM coupling constant, g, by sweeping the cavity through the triplet transition via a gas adsorption technique. The resulting time-photoluminescence map in Supplementary Fig. 2 exhibits a clear anticrossing. The minimum separation between the peaks (ΔE) at the anticrossing is ~80 μeV and is related to g by[3]

$$\Delta E = 2\sqrt{g^2 - \frac{(\Gamma_\mathrm{T}-\kappa)^2}{16}}, \qquad (1)$$

where κ = 190 μeV is the cavity linewidth and Γ_T = 28 μeV is the triplet linewidth estimated from Supplementary Fig. 3b. Using this equation we find that $g \approx 57$ μeV and therefore our cavity-QDM system is just in the strong coupling regime ($g > \kappa/4 \approx 47.5$ μeV). In addition, note the enhancement of the anti-Stokes Raman as the cavity is swept through it.

Supplementary Note 3. Bias-dependent Raman linewidth

A single photon source based on a Raman process has many advantages over other schemes, one of which is that the linewidth of the photon is determined by the spin-dephasing time, not the spontaneous emission lifetime. However, utilizing a scanning Fabry-Perot interferometer we observe relatively broad Raman photons (~11 μeV) compared to earlier work with a single spin (3 μeV).[4] There are number of processes which could cause this effect, one of which is fast spin-relaxation due to the large exchange energy.[5] The Raman linewidth can also be increased by electric field fluctuations in either the voltage source or the local environment that cause jitter in the singlet, triplet, and excited state energy levels. Such spectral wandering effects can be significant in QDMs as the transition energies strongly depend on bias.

To test this, we extract the singlet, triplet, and anti-Stokes Raman full width at half maximum (FWHM) linewidths from the data in Fig. 3a as a function of bias. Example one Lorentzian (used for the singlet and triplet) and two Lorentzian (used for the cavity-stimulated Raman) fits to the data are shown in Supplementary Fig. 3a, clearly demonstrating that the Raman linewidth can vary with sample bias. The full dependence of the Raman linewidth on bias is shown in Supplementary Fig. 3b and the variation is significant, reaching a minimum value at the "sweet spot" ($\frac{dE_{\mathrm{ex}}}{dV} = 0$). The singlet and triplet transitions also exhibit variations, although they are less severe. We find that we can fit the bias-dependent Raman linewidth with the following model that accounts for voltage fluctuations of the exchange energy:

$$\Gamma_{\mathrm{aSR}}^2(V) = \left(\Delta V \left|\frac{dE_{\mathrm{ex}}}{dV}\right| + \frac{1}{2}\Delta V^2 \left|\frac{d^2E_{\mathrm{ex}}}{dV^2}\right|\right)^2 + \Gamma_0^2 + \Gamma_{\mathrm{intrinsic}}^2. \quad (2)$$

Γ_{aSR} is the experimentally measured anti-Stokes Raman linewidth, ΔV is the amplitude of the voltage fluctuations experienced by the sample, E_{ex} is the bias-dependent exchange energy, Γ_0 is the spectrometer resolution, and $\Gamma_{\mathrm{intrinsic}}$ is the intrinsic Raman linewidth. The fit to the data is excellent (Supplementary Fig. 3b) when we use values of $\Delta V = 4.5$ mV (a value consistent with observations on past QDM samples), $\Gamma_0 = 15$ μeV (the spectrometer resolution), and $\Gamma_{\mathrm{intrinsic}} =$ 8 μeV. The extracted value of $\Gamma_{\mathrm{intrinsic}}$ agrees well with our Fabry Perot measurements suggesting that while electric field fluctuations do play a role, there are additional mechanisms broadening the Raman linewidth. It may be possible to reduce Raman linewidth by decreasing the exchange splitting. This will both extend the voltage range over which $\frac{dE_{\mathrm{ex}}}{dV} \approx 0$ and also reduce the spin dephasing rate arising from cotunneling to the electron reservoir.

Supplementary Note 4. Raman power dependence

In Fig. 4b of the manuscript we plot the integrated intensity of the cavity-stimulated and resonant anti-Stokes Raman at a single laser power. In Supplementary Fig. 4 we present a complete characterization of the effect of laser power on the peak intensity, linewidth, and integrated intensity of the anti-Stokes Raman. These values are extracted by fitting a sum of three Lorentzians to the data. Supplementary Fig. 4a shows the Raman peak intensity for the resonant case (solid markers) and the cavity-stimulated case (open markers) at three detunings. Signs of saturation are evident in the resonant condition but less-so for the cavity-assisted process. We observe that at high powers the peak intensity of the cavity-assisted Raman can actually become comparable to resonant Raman, even at a cavity detuning of Δ_{CS} = -400 μeV. Finally, we note that the Raman intensities in Fig. 3 and Supplementary Fig. 4 differ slightly. We attribute this to variations in the collection efficiency as these data were acquired on different days.

The Raman linewidth exhibits a surprising dependence on the laser power (Supplementary Fig. 4b). This is particularly pronounced for the resonant cases where the linewidth increases as the laser power exceeds ~10 μW for all cavity detunings. In addition, Raman photons for the doubly resonant case (Δ_{CS} = 0 μeV) appear to have a larger linewidth than the detuned cases. The linewidth of cavity-stimulated Raman photons increases only marginally.

Finally, while the peak intensity saturates for the resonant Raman, the integrated intensity does not (Supplementary Fig. 4c). This occurs because of the substantial broadening of the resonant Raman photons. Why this broadening is not compensated for by a concomitant reduction in peak intensity remains an open question.

Supplementary Note 5. Expressions for the Autler-Townes and AC Stark effects

The Autler-Townes effect can most easily be described in a strongly-driven two-level system. If the drive field is made sufficiently strong, the levels become “dressed” by the laser field and form polariton-like states. As a result both the ground (E_{g}) and excited (E_{e}) states split, forming a pair levels separated by the generalized Rabi frequency,

$$E_{\mathrm{g}} = E_{\mathrm{g}}^{(0)} - \frac{\delta}{2} \pm \frac{sgn(\delta)}{2}\sqrt{\Omega_0{}^2 + \delta^2}, \tag{3}$$

$$E_{\mathrm{e}} = E_{\mathrm{e}}^{(0)} + \frac{\delta}{2} \pm \frac{sgn(\delta)}{2}\sqrt{\Omega_0{}^2 + \delta^2}. \tag{4}$$

Ω_0 is the Rabi frequency of the laser, δ is the laser detuning from the transition, and $E_{i=\mathrm{g,e}}^{(0)}$ corresponds to the unperturbed state energies. This splitting can be directly resolved in a three-level system by observing emission or absorption between the dressed excited states and a spectator state. The spectral signature is a doublet spilt by Ω_0 on resonance (δ=0).

In the limit of large detuning ($\Omega_0/\delta \ll 1$) the eigenstates are well described by the bare states, which are weakly dressed and experience small energy shifts. The magnitude of this shift can be found by expanding Eqs. (3,4) to second order in Ω_0/δ and keeping only the solution close to $E_{i=\mathrm{g,e}}^{(0)}$. This results in

$$E_{\mathrm{g}} - E_{\mathrm{g}}^{(0)} = \frac{1}{4}\frac{\Omega_0{}^2}{\delta}, \tag{5}$$

$$E_{\mathrm{e}} - E_{\mathrm{e}}^{(0)} = -\frac{1}{4}\frac{\Omega_0{}^2}{\delta}. \tag{6}$$

and is known as the AC Stark shift. Note that the amplitude of the shift scales linearly with the laser *intensity*, in contrast to the Autler-Townes splitting.

Since the cavity is strongly driven, we anticipate that a good approximation is to ignore the effect of the QDM on the cavity which is weak compared to the laser. The steady state of the cavity can then be modeled as an effective field driving the QDM. Following this process we find that the Rabi frequency appearing in Eqs. (3-6) is replaced by a cavity-enhanced Rabi frequency:

$$\Omega = \Omega_0 + g \frac{\Omega_\mathrm{C}}{\sqrt{\delta_\mathrm{C}^2 + \frac{\kappa^2}{4}}}. \tag{7}$$

Here, Ω_C is the coupling of the laser to the cavity and δ_C is the detuning of the laser from the cavity (Fig. 2b). We find that these laser-cavity interactions cause remarkable modifications of the Autler-Townes and AC Stark effects as shown in Fig. 5 and Fig. 6 of the manuscript.

Supplementary Note 6. Cavity-QDM modeling

When the system is driven by vertically polarized light the cavity is driven very strongly. The interaction of the cavity with the external field is much stronger than the interaction of the cavity with the quantum dot, which to first approximation we can ignore. Our strategy is therefore to solve the equation of motion of the cavity annihilation/creation operator for the driven and lossy cavity in the absence of the quantum dot. This leads to a steady state solution for the cavity operators which can then be plugged into the equation of motion for the quantum dot degrees of freedom, allowing us to obtain an effective Hamiltonian for the QDM alone. This approximation is justified because there is an asymmetry in how strongly the QDM affects the cavity versus how much the cavity affects the QDM. It should be a valid approximation in the limit where the cavity is populated with a large number of photons, $N \gg 1$. Following this process we derive an effective cavity-induced driving of the QDM, effectively amplifying the driving field the quantum dots experience.

The Hamiltonian of the system is

$$H = H_0 + H_{QD-L} + H_{QD-C} + H_{C-L}, \tag{8}$$

where the terms respectively describe the QDM and cavity system in the absence of interactions, the interaction between the QDM and the laser, the interaction between QDM and cavity, and the interaction between the cavity and the laser. The first three terms of the Hamiltonian contain contributions from the 4-level singlet-triplet subspace as well as the 2-level T_+ and T_- transitions (Fig. 2b):

$$H_0 = H_{0,ST} + H_{0,+} + H_{0,-} + \omega_\mathrm{C}\, a^+ a \tag{9}$$

$$H_{QD-L} = H_{QD-L,ST} + H_{QD-L,+} + H_{QD-L,-} \quad (10)$$

$$H_{QD-C} = H_{QD-C,ST} + H_{QD-C,+} + H_{QD-C,-} \quad (11)$$

The ST, $+$, and $-$ subscripts indicate the singlet-triplet subspace, the T_+ transition, and the T_- transition, ω_{C} is the cavity frequency, and a, a^+ are the annihilation and creation operators for the cavity. The specific form of each of these terms is as follows:

$$H_{0,ST} = E_{\mathrm{T}} |T_0\rangle\langle T_0| + E_1|X_1\rangle\langle X_1| + E_2|X_2\rangle\langle X_2| \quad (12)$$

$$H_{0,+} = E_{\mathrm{T}} |T_+\rangle\langle T_+| + E_+|X_+\rangle\langle X_+| \quad (13)$$

$$H_{0,-} = E_{\mathrm{T}} |T_-\rangle\langle T_-| + E_-|X_-\rangle\langle X_-| \quad (14)$$

$$H_{QD-L,ST} = \Omega_V(t)(|T_0\rangle\langle X_1| + |S\rangle\langle X_2|) + \Omega_H(t)(|T_0\rangle\langle X_2| + |S\rangle\langle X_1|) + h.c. \quad (15)$$

$$H_{QD-L,+} = \frac{1}{\sqrt{2}}\Omega_V(t)|T_+\rangle\langle X_+| + \frac{1}{\sqrt{2}}\Omega_H(t)|T_+\rangle\langle X_+| + h.c. \quad (16)$$

$$H_{QD-L,-} = \frac{1}{\sqrt{2}}\Omega_V(t)|T_-\rangle\langle X_-| + \frac{1}{\sqrt{2}}\Omega_H(t)|T_-\rangle\langle X_-| + h.c. \quad (17)$$

$$H_{QD-C,ST} = g(|T_0\rangle\langle X_1| + |S\rangle\langle X_2|)a^+ + h.c. \quad (18)$$

$$H_{QD-C,+} = \frac{1}{\sqrt{2}}g|T_+\rangle\langle X_+|a^+ + h.c. \quad (19)$$

$$H_{QD-C,-} = \frac{1}{\sqrt{2}}g|T_-\rangle\langle X_-|a^+ + h.c. \quad (20)$$

$$H_{C-L} = \Omega_{\mathrm{C}}(t)a^+ + \Omega_{\mathrm{C}}^*(t)a. \quad (21)$$

All the energies in the Hamiltonian are measured from the singlet, which defines the zero of energy. In these expressions, E_{T} is the energy of the T_0, T_+, and T_- triplets, E_1 is the energy of the trion state $|X_1\rangle$, E_2 is the energy of the trion state $|X_2\rangle$, E_+ is the energy of the trion state $|X_+\rangle$, E_- is the energy of the trion state $|X_-\rangle$, $\Omega_J(t)$is the Rabi frequency of vertically (*J*=*V*) and horizontally (*J*=*H*) polarized transitions of the QDM, g is the coupling to the cavity, and $\Omega_{\mathrm{C}}(t)$ is the Rabi frequency of the cavity.

Moving to the rotating frame of the pulse and defining the detuning of the cavity from the laser Δ_C, the equation of motion for the annihilation operator is $\dot{a} = -i\,\Delta_{\mathrm{C}}a - \frac{\kappa}{2}a - i\,\Omega_{\mathrm{C}}$ where κ is the cavity loss rate. The steady state solution is then

$$a = \frac{-i\,\Omega_{\mathrm{C}}}{i\,\Delta_{\mathrm{C}} + \kappa/2} \quad (22)$$

Moving to the lab frame and plugging Eq. (22) into the term H_{QD-C} we finally find for the effective Hamiltonian of the QDM:

$$H_{eff} = H_0 - \omega_C\, a^+ a\; + H_{QD-L} + \left[\frac{i\, g\, \Omega_C(t)}{-i\, \Delta_C + \frac{\kappa}{2}} (|T_0\rangle\langle X_1| + |S\rangle\langle X_2|) + h.c.\right] +$$

$$\frac{1}{\sqrt{2}}\left[\frac{i\, g\, \Omega_C(t)}{-i\, \Delta_C + \frac{\kappa}{2}} (|T_+\rangle\langle X_+|) + h.c.\right] + \frac{1}{\sqrt{2}}\left[\frac{i\, g\, \Omega_C(t)}{-i\, \Delta_C + \frac{\kappa}{2}} (|T_-\rangle\langle X_-|) + h.c.\right]. \tag{23}$$

The last three terms in brackets corresponds to the cavity-enhanced drive of the quantum dot system. Grouping the terms describing vertically polarized transitions and dropping the phase, we find an effective Rabi frequency for all vertically polarized QDM transitions in the singlet-triplet subspace which equals

$$\Omega_{eff,ST}(t) = \Omega_V(t) + \frac{g\, \Omega_C(t)}{\sqrt{\Delta_C^2 + \frac{\kappa^2}{4}}} = \Omega_V(t)\left(1 + \frac{g\, \Omega_C(t)/\Omega_V(t)}{\sqrt{\Delta_C^2 + \frac{\kappa^2}{4}}}\right). \tag{24}$$

A similar expression can be obtained for the T_+, and T_- transitions:

$$\Omega_{eff,\pm}(t) = \Omega_V(t) + \frac{1}{\sqrt{2}}\frac{g\, \Omega_C(t)}{\sqrt{\Delta_C^2 + \frac{\kappa^2}{4}}} = \Omega_V(t)\left(1 + \frac{1}{\sqrt{2}}\frac{g\, \Omega_C(t)/\Omega_V(t)}{\sqrt{\Delta_C^2 + \frac{\kappa^2}{4}}}\right). \tag{25}$$

We can thus view the cavity as amplifying the Rabi frequency that the vertically polarized transitions of the QDM feel by a term equal to the quantity in the bracket. This model captures the cavity-enhanced excitation of the QDM very well, but does not include any cavity-enhanced emission processes.

We use the effective Hamiltonian derived above, as well as the standard Lindblad operators $L_{S,1} = \sqrt{\gamma}|S\rangle\langle X_1|$, $L_{S,2} = \sqrt{\gamma}|S\rangle\langle X_2|$, $L_{T_0,1} = \sqrt{\gamma}|T_0\rangle\langle X_1|$, and $L_{T_0,2} = \sqrt{\gamma}|T_0\rangle\langle X_2|$ for the optical relaxation, and $L_{S,T_0} = \sqrt{\Gamma}|S\rangle\langle T_0|$ for the relaxation between singlet and triplet states. $L_{T_+,+} = \sqrt{\gamma}|T_+\rangle\langle X_+|$ and $L_{T_-,-} = \sqrt{\gamma}|T_-\rangle\langle X_-|$ similarly describe optical relaxation of the T_+, and T_- transitions. We also include a pure dephasing term for the optical transitions, with rate γ_{deph}.

We solve for the resonance fluorescence signal for the system using the standard expressions

$$\Lambda_H(\omega) = Re\Big(\int_0^\infty d\tau\;\, e^{i\,\omega\,\tau}\big[\langle\sigma_{SX_1}^+(0)\sigma_{SX_1}(\tau)\rangle + \langle\sigma_{T_0X_2}^+(0)\sigma_{T_0X_2}(\tau)\rangle + \langle\sigma_{SX_1}^+(0)\sigma_{T_0X_2}(\tau)\rangle + \langle\sigma_{T_0X_2}^+(0)\sigma_{SX_1}(\tau)\rangle + \langle\sigma_{T_+X_+}^+(0)\sigma_{T_+X_+}(\tau)\rangle + \langle\sigma_{T_-X_-}^+(0)\sigma_{T_-X_-}(\tau)\rangle\big]\Big) \tag{26}$$

for horizontally polarized detection and

$$\Lambda_V(\omega) = Re\Big(\int_0^\infty d\tau\;\, e^{i\,\omega\,\tau}\big[\langle\sigma_{SX_2}^+(0)\sigma_{SX_2}(\tau)\rangle + \langle\sigma_{T_0X_1}^+(0)\sigma_{T_0X_1}(\tau)\rangle + \langle\sigma_{SX_2}^+(0)\sigma_{T_0X_1}(\tau)\rangle + \langle\sigma_{T_0X_1}^+(0)\sigma_{SX_2}(\tau)\rangle + \langle\sigma_{T_+X_+}^+(0)\sigma_{T_+X_+}(\tau)\rangle + \langle\sigma_{T_-X_-}^+(0)\sigma_{T_-X_-}(\tau)\rangle\big]\Big) \tag{27}$$

for vertically polarized detection. In these equations the symbol $\sigma_{JX_i}^{+}$ denotes the raising operator between state $|J\rangle = |S\rangle, |T_0\rangle, |T_+\rangle, |T_-\rangle$ and trion $|X_i\rangle, i = 1, 2, +, -$. The expectation value is taken with respect to the steady state and the quantum regression theorem is used to facilitate the calculation.

All parameters are taken from the experiment, with the exception of γ_{deph} and Ω_{C}, which are not known. These are adjusted so that the theoretical plots match the experimental data. Below we include a list of all the numbers used in our simulations.

$\Omega_J = 6.75$ μeV, $\gamma = 10$ μeV, $\gamma_{\mathrm{deph}} = 24$ μeV, $g = 57$ μeV, $\kappa = 186$ μeV, $\Omega_{\mathrm{C}} = 7.02$ meV, and $E_{\mathrm{T}} = 1.455$ meV.

Supplementary Note 7. Polarization dependence

We discuss in the text the leak-through of cavity polarized light in Fig. 5a and Fig. 6d This necessitates that our calculations include both *V* and *H* emission of comparable magnitude to capture all the spectral lines seen in the experiment. In Supplementary Fig. 5 we isolate the individual contributions of *V* and *H* emission. Supplementary Fig. 5a is identical to Fig. 5b, containing *V* and *H* contributions. Supplementary Fig. 5b contains only *H* emission, which includes contributions from the cross transitions 2 and 3. Supplementary Fig. 5c contains purely *V* emission, which contains contributions from transitions 1 and 4, and resembles the AC Stark shifts expected for a pair of two-level systems. An additional point we wish to address is the role of the T_+/T_- transitions. While these transitions do not show Autler-Townes splittings as they are not part of the Λ-configuration, they do experience AC Stark shifts of decreased magnitude since they are circularly polarized (Supplementary Fig. 5d). Therefore, there is the appearance of a third AC Stark shifted line around the triplet for both *H* and *V* polarized emissive components which is labeled as transition 5. This is not visible experimentally due to the broadening of the emission lines.

Supplementary Note 8. Control of the exchange energy

In the manuscript we discuss our ability to control the magnitude of the exchange energy through the cavity-enhanced AC Stark effect. This is well summarized by the data in Fig. 5e in the main text where we show E_{ex} as a function of laser energy for two detunings: Δ_{CS} = -980 μeV and Δ_{CS} = -1920 μeV. Here, we show the full spectral map for the second detuning, Δ_{CS} = -1920 μeV, in Supplementary Fig. 6a for the (*V*,*H*) polarization configuration. The cavity is red detuned from all transitions, forcing the singlet and triplet to blueshift as the laser is swept over the cavity mode. Since the cavity is closer to the triplet than the singlet, the triplet experiences a larger AC Stark shift and the exchange energy decreases. We have calculated the energies and emission intensities of the cavity-QDM system under these conditions and find good agreement between

theory (Supplementary Fig. 6b) and experiment (Supplementary Fig. 6a). We again note that while the agreement between the experimental and theoretical energies is excellent, there are additional variations in the measured emission intensity and linewidth not captured by our model.

Supplementary References

1. Scheibner, M., Bracker, A., Kim, D. & Gammon, D. Essential concepts in the optical properties of quantum dot molecules. *Solid State Commun.* **149,** 1427–1435 (2009).
2. Doty, M. *et al.* Optical spectra of doubly charged quantum dot molecules in electric and magnetic fields. *Phys. Rev. B* **78,** 115316 (2008).
3. Laucht, A. *et al.* Dephasing of Exciton Polaritons in Photoexcited InGaAs Quantum Dots in GaAs Nanocavities. *Phys. Rev. Lett.* **103,** 087405 (2009).
4. Sweeney, T. M. *et al.* Cavity-stimulated Raman emission from a single quantum dot spin. *Nat. Photonics* **8,** 442–447 (2014).
5. Elzerman, J. M., Weiss, K. M., Miguel-Sanchez, J. & Imamoğlu, A. Optical Amplification Using Raman Transitions between Spin-Singlet and Spin-Triplet States of a Pair of Coupled In-GaAs Quantum Dots. *Phys. Rev. Lett.* **107,** 017401 (2011).
